# Impact of the COVID-19 pandemic on care delivery, follow-up and outcomes in chronic myeloid leukemia patients: An observational cohort study

**DOI:** 10.6026/9732063002001813

**Published:** 2024-12-31

**Authors:** Pramod Kumar Pamu, Tara Roshni Paul, Naval Chandra, Sadashivudu Gundeti, Radhika K.

**Affiliations:** 1 Department of Pathology, Nizam's Institute of Medical Sciences (NIMS), Punjagutta, Hyderabad 500082, Telangana, India; 2 Department of Medicine, Nizam's Institute of Medical Sciences( NIMS), Punjagutta, Hyderabad 500082, Telangana, India; 3 Department of Medical Oncology, Department of Pathology, Nizam's Institute of Medical Sciences (NIMS), Punjagutta, Hyderabad 500082, Telangana, India

**Keywords:** Covid 19, chronic myeloid leukemia, care delivery

## Abstract

Chronic myeloid leukemia (CML) is a myeloproliferative neoplasm that comprises a chronic phase. Therefore, it is of interest to evaluate the impact of care delivery and loss of follow-up or defaulted treatment due to the COVID-19 pandemic affecting the outcome in known chronic myeloid leukemia patients. Data was retrospectively retrieved and prospectively evaluated from the known and treated CML patients. A questionnaire was prepared for history. Bone marrow slides stained with Giemsa stain and multi-color flow cytometry were used for the evaluation of blast type in all blast crisis cases. A total of 961 new CML cases were reported, age range from 21 to 78 years, results were analyzed in three different cohort groups based on their time of diagnosis. Loss of follow-up was noticed mainly during the COVID-19 period and thereafter because of non-compliance. Data shows that about 53% of cases showed no hematological response and about 34% transformed into a CML blast crisis phase.

## Background:

Chronic Myeloid Leukaemia (CML) is a model for modern precision medicine, where treatment is tailored to a patient's genetic profile. All CML patients exhibit the t (9:22) chromosomal translocation, resulting in the fusion of the BCR gene from chromosome 22 and the ABL kinase domain from chromosome 9. This genetic abnormality is crucial for diagnosing and classifying CML, which can be detected through cyto-genetics or RT-PCR. The BCR-ABL gene also plays a key role in monitoring the disease, helping assess treatment response. Imatinib mesylate, a tyrosine kinase inhibitor (TKI), specifically targets the BCR-ABL protein by inhibiting the ABL kinase activity, thereby preventing the proliferation of CML cells [1]. The advent of TKI therapy has dramatically improved survival rates, with the natural history of CML shifting from a life expectancy of about seven years to almost normal life expectancy. The COVID-19 pandemic, which emerged in late 2019, posed one of the greatest health threats to the current generation, overwhelming healthcare systems globally [2]. As the virus spread, healthcare facilities had to shift their focus from regular patient care to the emergency treatment of large numbers of COVID-19 patients. This redirection of resources disrupted essential care for many patients with chronic conditions, including those with CML, a blood and bone marrow cancer that requires continuous and systematic follow-up [3]. The management of CML relies on consistent laboratory monitoring, regular follow-ups with oncologists and strict adherence to TKI therapy. Unfortunately, the pandemic interrupted these vital healthcare services, leading to missed appointments and follow-ups, likely negatively affecting CML patients [4]. CML patients were affected directly when healthcare resources were redirected during the pandemic. Hospitals and clinics were under pressure to address COVID-19 cases, meaning individuals requiring other services such as checkups, tests and other illnesses vital treatments were either postponed or declined [5]. COVID-19 has significantly affected health service delivery around the globe. In March 2020, at the start of the COVID-19 outbreak, The Max Foundation was assisting over 32,000 patients under the supervision of 527 doctors in 72 LMICs. COVID-19 has inevitable effects on people in LMICs In the following aspects. The Max Foundation's MAS programs that offer people in need access to life-saving cancer medications were greatly affected by the pandemic. Dynamic solutions were employed at the patient, local, institutional and physician levels, the supply chain partners, headquarters levels and the staff at both strategic and operational levels. Some of the CC disruptions in global supply chains include airline shutdowns, flight cancellations, rejection of imported consignments and a long delay in permitting imports. This disruption meant that the CML patient community was at high risk of seeing the stocks of these medicines run out in-country, the medicines expiring and, most alarmingly, the early deaths of CML patients. Most of the CML patients, especially those with immunosuppression, reported heightened concerns and apprehension about contracting the virus, thus resulting in the delay or omission of clinical appointments [6]. Face-to-face contact was not fully replaced, but telemedicine became a popular practice and it was necessary to include it in further treatment of CML. For this reason, most patients received a disrupted cycle of treatment, delayed assessment for possible adverse effects and insufficient consistent check-ups [7]. The COVID-19 pandemic and its measures have impacted the lives of people, especially those who have had chronic conditions. Conditions like human immune-virus (HIV), diabetes mellitus (DM) and hypertension are immunosuppressing cases, which mean that patients are not just more susceptible to infection but are also likely to develop severe sickness from COVID-19. Furthermore, these patients with COVID-19 are less likely to be cured compared to other patients with other diseases [8]. For example, deaths caused by HIV over five years could be raised by the level of up to 10% compared to the situation before the COVID-19 pandemic in the heavily affected regions. The pandemic impacted comprehensive routine care for chronic patients since delivery care in various ways was interrupted.

Firstly, actions like lockdowns, shutting borders, quarantining, social distancing and community isolation affect the supply and access to Medications. Second, the number of patients is high because they are focused on COVID-19 patients. As a result, patients needing long-term follow-up delayed the follow-up. Hence, the pandemic contributes to panic, especially in the sub-Saharan region, where the health system is relatively intolerant of the disease [7]. As a result of COVID-19 measures such as physical distancing, which exacerbate loneliness, an individual with chronic health conditions with compromised immune systems may have a sterner stress response to COVID-19 than the overall population. Thus, they are obliged to forfeit follow-up from chronic disease care. Public health responses towards COVID-19 are distressing to PLWHs (Patient living with HIV) and interfere with their adherence to ART [8]. The published literature revealed that approximately 25 per cent of people in ART interrupted their treatment during the COVID-19 response period. Since the approximate final 'landed' cost of exported antiretroviral medicines is raised from pre-CSF rates, PLWHs on ART in India were compelled to suspend their treatment discretely. This disruption is even more worrisome to CML patients and even though the disease is relatively manageable in the modern world, with proper care, it demands it. TKIs, which constitute the mainstay of CML treatment, depend on the patient's compliance and frequent monitoring of the molecular response by BCR-ABL1 transcript quantification [8]. Failure to follow up can cause missed increases in the disease burden and/or resistance to treatment or state progression to a phase which is much harder to treat and the prognosis is poorer than in leukemia. Further, the pandemic stress and, thus, the panic may have equally led to poor compliance with medications and worsening follow-up perils associated with the illness [9-10]. Therefore, it is of interest to evaluate the impact of care delivery and loss of follow-up or defaulted treatment due to the COVID-19 pandemic affecting the outcome in known chronic myeloid leukemia (CML) patients.

## Methods and Materials:

The study was conducted over a total duration of 6.5 years, from January 2017 to June 2023. This period was selected to capture data before and after the COVID-19 pandemic, allowing for a comprehensive evaluation of its impact on the care delivery and outcomes in patients with Chronic Myeloid Leukemia (CML). This study employed a mixed retrospective and prospective observational design. Data were retrospectively retrieved from electronic medical records (EMRs) and prospectively evaluated in known cases of CML.

## Inclusion criteria:

[1] Known cases of CML in the chronic phase (CP) or accelerated phase (AP) on tyrosine kinase inhibitor (TKI) medication.

[2] Patients with a history of COVID-19 infection during or after the pandemic period.

[3] Cases that experienced a loss of follow-up and progressed to CML blast crisis or showed no haematological response on bone marrow aspiration (BMA) or flow cytometry study.

## Exclusion criteria:

[1] Cases diagnosed with CML in the blast crisis (BP) phase at initial diagnosis.

[2] Patients who were lost to follow-up immediately after their initial diagnosis.

[3] Patients who did not contract COVID-19 during the study period.

[4] Cases with incomplete clinical details in their medical records.

## Data collection:

Clinical data were meticulously extracted from the electronic medical records of CML patients, including demographic information, clinical presentation at diagnosis, treatment regimens and outcomes. A structured questionnaire was prepared to gather detailed patient histories, covering aspects such as the onset of symptoms, treatment adherence and the impact of the pandemic on follow-up care. Bone marrow samples from patients were analyzed using various laboratory techniques. Giemsa-stained bone marrow slides and trephine sections stained with Hematoxylin and Eosin (H & E) and reticulin stain were examined to assess fibrosis. Additionally, multi-color flow cytometry was utilized to evaluate the type of blasts (Myeloid or Lymphoid) in patients who progressed to blast crisis. The criteria for diagnosing CML in blast crisis were based on the presence of ≥20% blasts in the peripheral blood or bone marrow. The study population was divided into three cohort groups based on the timing of their CML diagnosis during the COVID-19 pandemic:

[1] Cohort 1: Patients diagnosed with CML before the pandemic (January 2017 to March 2020).

[2] Cohort 2: Patients diagnosed during the pandemic (March 2020 to December 2021).

[3] Cohort 3: Patients diagnosed after the pandemic (January 2022 to May 2023).

## Data analysis:

The collected data were analyzed to assess the impact of the COVID-19 pandemic on the clinical outcomes of CML patients, focusing on those who experienced a loss of follow-up during the pandemic. The analysis included comparisons of progression rates to blast crisis, hematological response and overall survival across the three cohorts.

## Results:

A total of 961 new CML cases were reported, with ages ranging from 21 to 78 years; results were analysed in three different cohort groups based on their time of diagnosis. The mean age of the patients was 38.4 years in the chronic phase and 46.4 years in the blast phase. Cohort 1, diagnosed before the pandemic, had 569 newly diagnosed CML cases with a median age of 42 years, predominantly male (21% female). In this group, 77% experienced deteriorating disease conditions during follow-up. Cohort 2, diagnosed during the pandemic, had fewer cases (108), with a higher female percentage (32%) and a median age of 52 years. This group saw 80% deterioration in disease conditions. Cohort 3, diagnosed after the pandemic, included 284 cases with 41% female representation and a median age of 44 years (Table 1). Of 829 diagnosed cases, 280 (33.79%) remained on regular treatment, while 430 (44.79%) were classified as loss of follow-up cases. Among the regularly treated patients, 78.6% presented with an abdominal mass, 60.0% with loss of appetite and 60.1% with splenomegaly. In contrast, the loss of the follow-up group showed a significant reduction in these symptoms, with 28.6% reporting an abdominal mass, 26.0% loss of appetite and 21.0% splenomegaly. Bone marrow fibrosis was notably higher in the loss of follow-up group at 80.5%, compared to 23.7% in the regularly treated patients. Additionally, 27.0% of the loss of follow-up cases showed nil or partial haematological response, while 5.7% exhibited extra-medullary involvement (Table 2). The marrow findings indicate distinct differences between newly diagnosed CML cases and defaulters. Among the 829 newly diagnosed patients, 54% had particulate marrow, 90% showed marked myeloid prominence and 10% had marrow eosinophilia, with an average blast percentage of 2%. In contrast, defaulters exhibited more severe marrow abnormalities, with only 21% showing particulate marrow, 80% presenting with marrow fibrosis and a higher average blasts percentage of 10%, reflecting a more advanced disease state. The present consisted of 163 male and 32 female participants. Among the males, 101 experienced a myeloid blast crisis, 44 had a lymphoid blast crisis, 6 had a monocytic type blast crisis, 6 had bi-phenotypic leukemia and 6 showed extra-medullary involvement. Among the females, 18 experienced a myeloid blast crisis, 10 had a lymphoid blast crisis and 4 had a monocytic type blast crisis, with no cases of bi-phenotypic leukemia or extra-medullary involvement reported (Table 3).

Patients often presented with some history of symptoms such as fullness in hypochondrium, generalized weakness and other non-specific complaints. Specific details were elicited through all patients and their common features were the non-existence of significant gum bleeding, loss of body weight and gastrointestinal disorders. On first presentation, a full diagnostic workup was made on the patients, starting with bone marrow aspiration and biopsy to confirm the phase of CML. Polymerase chain reaction (PCR) tests for IRMA, as well as quantitative assays of BCR-ABL levels, were performed for assessing disease burden and observing treatment response. Patients being diagnosed with accelerated phase CML (CML-AP) usually had greater than 10% bone marrow blasts, as mentioned in the diagnostic tests. Treatment was started individually for each patient, depending on his or her profile and response to therapy. Imatinib was the drug of first choice, with the doses adjusted according to tolerance and efficacy. In cases of resistance or suboptimal response, as indicated by persistent high BCR-ABL levels or hematologic abnormalities such as neutropenia and thrombocytopenia, treatment was escalated to second-generation TKIs like Nilotinib or Dasatinib. Mutation analysis, including IRMA, was conducted to identify the resistance mutations such as G250E, which in turn guided the choice of alternative TKIs. Hematologic remission, defined as complete hematologic remission (CHR), was another frequent first milestone after initiation of therapy. Serial assessments of BCR-ABL allowed insight into molecular response and revealed that some patients demonstrated an initial decrease that later plateaued or rose. Therapy was adjusted for some of these patients based on this pattern. Suboptimal response to Dasatinib at 100 mg/day often required increasing the dose to 140 mg/day, especially if patients had not achieved significant enough reductions in BCR-ABL.

Peripheral smear and bone marrow aspirate/trephine biopsies were always conducted for investigations of cellularity of marrow, the presence of fibrosis as determined by reticulin staining and lineage-specific markers (for example, NSE staining for cells of the monocyte/macrophage lineage). Such an investigation will ensure an overall appraisal of disease course and the nature of the response to treatment. During follow-up, complications such as high LDH levels were monitored, especially post-transplantation, along with routine assessments of renal and liver function tests. Hemograms provided critical insights into hematologic parameters, aiding in identifying cytopenias or blast counts suggestive of disease activity. Figure 1(see PDF)illustrate the flow cytometry analysis in CML-blast crisis (myelomonocytic type), highlighting cell populations through marker expression. Positive markers include CD45, CD34, CD7, HLA-DR and CD64, indicating specific cellular features associated with blast crisis. Negative markers such as TdT, CD19 and CD15 help refine the diagnosis by showing markers not expressed in these cells. This analysis aids in understanding the cellular profile in CML-blast crisis.

## Discussion:

The study's results provide significant insights into the challenges CML patient's face, particularly those who experienced a loss of follow-up during the COVID-19 pandemic. Many patients were moving to a worse state, including blast crisis, which was regrettably noted in 195 patients, which could only emphasize the need to closely monitor and follow through on treatment regimens [11]. Eventual partial or nil haematological and molecular response was worryingly observed way back in 36 and 120 patient cases, respectively and all this makes it clear that discontinued treatment repeatedly up and over again not only raises the risk but shortfalls in subsequent follow-ups no matter how minimal will drastically reduce patient's prospects [12, 13-14]. Fibrotic changes in the bone marrow were detected in 32% of cases, as coagulative necrosis and infiltration of tissues with eosinophil's indicative of high disease activity in patients who do not adhere to medical treatment regimens. Such an untreated rate of CML patients in the grassroots population will mean that always, several patients will always default in their treatments and this will be worse given the global COVID-19 outbreak that affects consistent medical check-ups [15, 16-17]. There are also gender disparities, with more male participants presenting with severe complications, including myeloid blast crisis and extra medullary involvement, than female participants. It may indicate the development of differential follow-up plans by patient characteristics to enhance care provision [18]. The results stress the importance of having strong healthcare systems capable of maintaining chronic disease care irrespective of the context and ensuring the patient loses no chance for proper treatment that could have negative consequences [19]. The results of the present study correlate well and build further on prior literature about the distribution of blast crisis types in patients with CML [20, 21, 22- 23].

## Conclusion:

The outcome of CP-CML was adversely affected due to the pandemic period. Many patients lost their follow-up for TKI therapy whilst COVID-19 positive did appear to worsen the outcome. Thus, the COVID-19 pandemic had a significant negative impact on the outcomes of patients with Chronic Phase Chronic Myeloid Leukemia (CP-CML). This is particularly due to the disruption in follow-up for Tyrosine Kinase Inhibitor (TKI) therapy.

## Figures and Tables

**Table 1:** Characteristics of chronic myeloid leukemia (CML) patients

	**Diagnosed before the Pandemic (Cohort -1)**	**Diagnosed during the Pandemic (Cohort -2)**	**Diagnosed after Pandemic (Cohort -3)**
Duration	**2018 - March 2020**	**March 2020 - Dec - 21**	**January 2022 - May 2023**
Newly diagnosed cases of - CML	569	108	284
Female (Male preponderance)	121(21%)	35(32%)	101(41%)
Age years, median (IQR)	42 (21-74)	52 (38-78)	44 (36-64)
CML - CP Chronic Phase at diagnosis	501	85	243
CML - AP Accelerated phase	45	16	18
CML - BP Blastic pause	23	7	23
TKI at start of diagnosis N (%) Imatinib ≥2nd generation TKIa	510(89.5%)	60(55.5%)	250(88%)
	59 (10.5%)	25(44.5%)	34(11.8%)
Loss of follow-up during & after COVID-19 period	303	62	65
Follow-up years, median	4.3 (1-5.4)	1.3 (1-2.4)	0.9(1.-1.6)
History of COVID-19 infection	102	65	21
Vaccinated (two or three doses)	530	102	283
Loss of follow-up in cases	303	62	65
Worsening of disease on follow-up	234(77%)	49(80%)	33(50%)

**Table 2:** Presenting complaints in all groups

**Clinical & Molecular and BMA Responses**	**Diagnosed & on Regular Treatment (All Cohort Groups)**	**Loss of Follow-Up Cases**
Total Cases	n = 280/829 (33.79%)	n = 430/961 (44.79%)
Abdominal Mass	220 (78.6%)	122 (28.6%)
Loss of Appetite	170 (60.0%)	117 (26.0%)
Splenomegaly	167 (60.1%)	93 (21.0%)
Lymphadenopathy	-	17 (4.2%)
Primary TKI	216 (77.2%)	-
Secondary TKI	65 (23.5%)	-
BCR-ABL Status Testing	251 (89.7%)	-
Bone Marrow Fibrosis (Grade 2 or >2 Reticulin Condensation)	65 (23.7%)	339 (80.5%)
Extramedullary Involvement	-	6 (5.7%)
Nil or Partial Hematological Response	-	120 (27.0%)

**Table 3:** Bone marrow changes comparison in all groups

**Marrow Findings**	**At the Time of Diagnosis (New CML Cases)**	**% of Cases**	**In Defaulters' Cases**	**% of Cases**
Particulate Marrow	Yes	54%	Yes	21%
A Particulate with Hemodilution	Yes	25%	Yes	25%
A Particulate (Dry Tap)	-	-	Yes	54%
Marked Myeloid Prominence	Yes	90%	Yes	50%
Marrow Eosinophilia	Yes	10%	Yes	20%
Marrow Fibrosis	-	-	Yes	80%
Blasts Percentage (Avg.)	Yes	2%	Yes	10%
Lymph Plasmacytosis	Yes	1%	-	-
Reticulin Stain	Grade 1	-	-	-
Stromal Changes	-	-	Yes	21%
Hypocellular	-	-	Yes	28%
Total Cases	829	-	430	-
